# 

**Published:** 1890-08

**Authors:** 


### HALL'S JOURNAL OF HEALTH

ESTABLISHED 1854

CONTENTS.

VOL. 37. No. 8.

| | PAGE. |
|---|---|
| Mesmerism | 169 |
| Torpid Liver | 171 |
| The Occult Powers | 173 |
| The Proper Weight | 174 |
| The Stenographic Art | 175 |
| Tea and Coffee | 176 |
| An Athlete's Diet | 178 |
| Quinine Intoxication | 179 |
| Extracting Foreign Bodies from the Stomach | 179 |
| Born-Blindness Preventable | 180 |
| Poisoning by Tinned Fruit | 180 |
| The Tongue | 181 |
| Moths | 181 |
| Kindness to Animals | 182 |
| The Art of Prolonging Life | 184 |
| The Spread of Consumption | 184 |
| A Boy's Temper | 185 |
| Miscellaneous | 186 |
| The Mystery of the Magnet | 186 |
| Goldfish have fun with the Turtle | 187 |
| An Indian's Argument for Vegetarianism | 188 |
| Woman | 190 |
| Literary | 190 |
| Tell Me, My Heart | 192 |

INDEX TO ADVERTISEMENTS OF HALF A PAGE AND OVER.

| | PAGE. |
|---|---|
| Woman's Work | I |
| Celluloid | II |
| Hollow Suppositories | III |
| The Esoteric | IV |
| Hospital Remedy Co. | IV |
| Hall's Journal of Health | V |
| Goldsmith's Pianos | V |
| E. C. Morris & Co | VI |
| Dennin's Certain Cure | VII |
| The Victor Safe and Lock Co | VII |
| Revised Premium List | VIII |
| Herba-Vita Remedy Co. | IX, X |
| Christ Before Pilate | XI |

